# Supplementary material for: How many patients do you need? Investigating trial designs for anti‐seizure treatment in acute brain injury patients
Source: Ann Clin Transl Neurol. 2024 Jun 12;11(7):1681–90. doi: 10.1002/acn3.52059 (PMC11251465; doi:10.1002/acn3.52059)
Supplement: Supplementary file 1 — Table S1. Descriptive analysis and data summary of the cohort of patients in observational study. Figure S1. Histogram of mean squared errors for predicting the EA burden over time compared with the observed EA burden. The MSE is measured for each patient in the observational study. Figure S2. The ROC curve for the outcome prediction function. [file ACN3-11-1681-s001.docx]

**Supplementary Material** for

“How Many Patients Do You Need? Investigating Trial Designs for Anti-Seizure Treatment in Acute Brain Injury Patients”

# Notations

Our observational study cohort consists of aneurysmal subarachnoid hemorrhage (aSAH) patients from Massachusetts General Hospital (MGH). For each patient $i$ in the cohort, we collected pre-treatment covariates ($X_{i}$) including demographic features (such as age, sex, race, etc.), medical history (such as the history of epilepsy, chronic kidney disease, coronary artery disease, etc.), and the medical diagnosis during the time of admission. As aSAH patients can have seizures and other epileptiform activity (EA), every patient was under EEG monitoring. We used the EEG signals in a 10 minutes window at time $t$ to estimate the EA burden -- defined as the proportion of time the patient experienced EA in a given window. We denote the EA burden at time $t$ in the patient $i$ as $E_{it}$. Further, the ASMs administered at time $t$ in the patient $i$ are denoted by vector $W_{it}$ where the j-th entry denotes the drug dose in mg/kg. Finally, for each patient, we record the post-discharge outcome $Y_{i}$ using the binarized mRS, such that $Y_{i}=1$ if mRS for patient $i$ is greater than 3.

# Cohort Details

# Per institutional protocol all aSAH patients with Hunt and Hess scores >3 and Fisher scores >3 undergo 10 days of cEEG monitoring for ischemia detection. From our initial database of 136 patients with aSAH, we considered a subset of 48 patients who underwent more than 24 hours of EEG and had EA during monitoring. Per institutional protocol, all patients received levetiracetam until aneurysm was secured. The aneurysm is typically treated within the first 24 hours of admission, and primary prophylaxis discontinued immediately upon aneurysm treatment. Thereafter, levetiracetam or any other ASM treatment is continued at the discretion of the treatment team. Supplemental Table 1 shows the difference in baseline clinical and demographic covariates between patients that received ASM treatment vs. those that did not.

## Supplemental Table 1.

| Variable | ASM treatment  N= 32 | No ASM treatment  N= 16 | p-value |
| --- | --- | --- | --- |
| Age, median [Q1-Q3] | 59 [50-79] | 65 [56-71] | 0.443 |
| Sex, Female, N(%) | 24 (75%) | 14 (88%) | 0.271 |
| Hunt and Hess  1  2  3  4  5 | 2 (6%)  5 (16%)  10 (31%)  8 (25%)  7 (22%) | 4 (25%)  1 (6%)  4 (25%)  7 (44%)  0 (0%) | 0.07 |
| Fisher  2  3  4 | 1 (3%)  23 (72%)  8 (25%) | 1 (6%)  11 (69%)  4 (25%) | 1.000 |
| Initial GCS | 8 [4-14] | 8 [6-15] | 0.3939 |
| History of epilepsy | 1 (3%) | 0 (0%) |  |
| Seizure at ictus | 3 | 0 (0%) |  |
| Cardiac arrest | 1 | 0 (0%) |  |
| EEG findings  Seizures  Periodic/Rhythmic patterns | 4 (13%)  32 (100%) | 0 (0%)  16 (100%) |  |

# Methodology

In this section, we formally discuss the framework we employ to simulate RCTs. First, we discuss the simulator estimation procedure using the observational data which is divided into parts: (i) mechanistic pharmacological modeling and (ii) outcome modeling. Second, we discuss the procedure for simulating hypothetical randomized control trial using the learned models.

## Mechanistic Pharmacological Modeling

We modeled the short-term effect of ASMs on EA using a mechanistic pharmacokinetics-pharmacodynamics (PK/PD) model. Using mechanistic models guarantees the interpretability of the estimates as the parameters of the mechanistic model have physical interpretation and are well-suited for our application as they require lesser data to calibrate compared to an empirical statistical model.

**Pharmacokinetics.** We use model pharmacokinetics using a one-compartment model to estimate the concentration of drugs over time for any patient:

$$D_{ijt}=W_{ijt}+e^{-\gamma_{ij}}D_{ijt-1}$$

**Pharmacodynamics.** We use Hill’s pharmacodynamics model to estimate the short-effectiveness of drugs as a function of drug-concetrations for any patient:

$$E_{i,t}=E_{i,max}\left( 1-\sum_{j} \frac{D_{ijt}^{\alpha_{ij}}}{D_{ijt}^{\alpha_{i}}+ED{50}_{ij}^{\alpha_{ij}}} \right)$$

## Outcome Modeling

Parikh et al. (2022) showed that the maximum EA burden in a sliding 6 hours window has a significant negative effect on the post-discharge outcome, denoted by $E^{max}$. Following this result, we focus on modeling $Y$ as a function of $E^{max}$ and average ASM concentration during the course of treatment, $D^{mean}$. $Y_{i}(e,d)$ refers to the potential outcome if unit $i$ had $E_{i}^{max}=e$ and $D_{i}^{mean}=d$. We use gradient boosting trees based doubly machine learning (GBT-DML) to estimate $\boldsymbol{E}[Y\left( e,d \right)]$ -- the long-term effect of EA burden and ASMs on the post-discharge outcome. We adjust for patients' pre-treatment covariates as well as their PK/PD parameters.

## Simulating RCT

We designed trials to estimate the effectiveness of varying treatment regimens for a commonly prescribed ASM, levetiracetam, and a commonly prescribed anesthetic, propofol. We used the estimators learned using the observational data to perform an in-silico RCT by sampling and simulating patients' courses in the ICU. We analyzed how the required sample size and the associated power varied with choice of treatment arms and outcomes.

For each treatment arm, we sampled N units from the learned joint distribution of parameters and covariates via observational study. Next, we simulate each patient's ICU trajectory of EA burden as a function of the treatment regime. For instance, a patient in t-hours delay arm with d-dose of Levetiracetam will be administered their first dose of d strength t-hours after the admission to ICU. The subsequent doses of levetiracetam are administered at a gap of 8 hours. Unlike levetiracetam, propofol is not administered as a bolus but rather as an infusion. Thus, for propofol, we simulate the trajectory with a constant infusion of d-dose per 10 minutes. Finally, we compute the expected outcomes - $E^{max}$, $E^{mean}$, and Y - for each of the treatment arms using N simulated samples. We compare the outcomes from each treatment arm with the outcome from the placebo arm and compute the power of this analysis. Thus, varying the sample size N and re-performing the analysis allows us to identify the smallest sample to achieve a power of more than 80% for each treatment arm and outcome.

# Model Performance

Now, we present the performance of our methods on the observational data (which is also used to learn these models.) The choice of using the same data for training and evaluating model performance is due to the small sample size of the observational study. In what follows, we show the goodness of fit for the pharmacological models jointly as well as the outcome model. While each of the are essentially causal models targeting the potential outcomes values, we assess if they can predict the respective quantities under the observed treatment choices.

For assessing the performance of pharmacological models, we compare the observed EA burden ($E_{it}$) with the EA burden simulated using the learned pharmacological models ($\hat{E}_{it}$) under the observed drug regime. For each patient, we calculate the MSE = $\frac{1}{T_{i}}\sum_{t} \left( E_{it}- \hat{E}_{it} \right)^{2}$ where $T_{i}$ is the length of patient i's stay. Figure 1 shows the distribution of MSE across the observational cohort. The average MSE is 0.067. The low value of MSE highlights the goodness of the fit for the mechanistic pharmacological model.


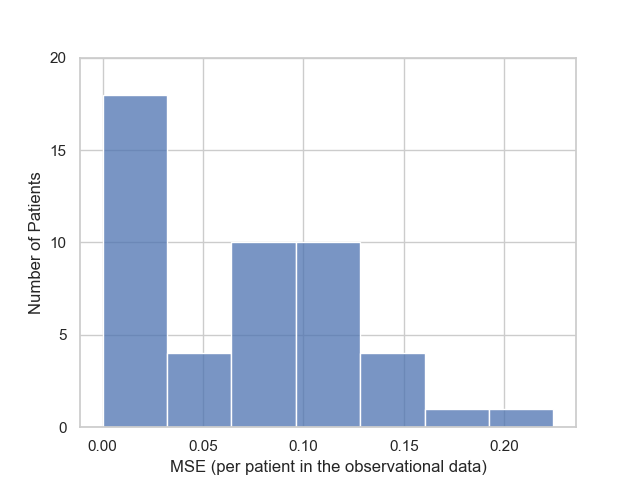


Figure 1. Histogram of Mean Squared Errors for Predicting the EA burden over time compared with the observed EA burden. The MSE is measured for each patient in the observational study.

Similarly, we compare the observed binary outcome and the predicted outcome under the observed drug dose and EA burden. The accuracy of our outcome model is 87.80%. Figure 2 shows the corresponding receiver operating characteristic curve for various levels of thresholds changing the true positive and false positive rates.


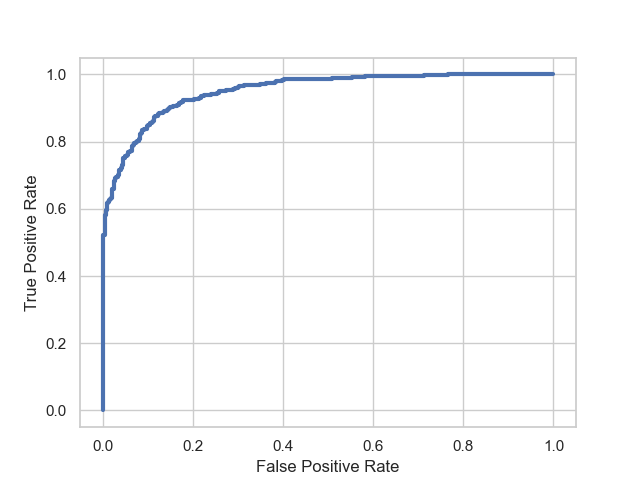


Figure 2. The ROC curve for the outcome prediction function.
